# Enhanced Seed Protein Yield in Hydroponically Grown Rice via Silica Hydrogel Application

**DOI:** 10.3390/plants15121775

**Published:** 2026-06-09

**Authors:** Yoshitomi Kudo, Kaede C. Wada, Akimasa Nakano, Koji Baba, Manami Furuya, Yuhya Wakasa, Jun-ichi Yonemaru

**Affiliations:** 1 Research Center for Agricultural Information Technology, National Agriculture and Food Research Organization, 1-31-1 Kannondai, Tsukuba 305-0856, Ibaraki, Japan; wada.kaede362@naro.go.jp (K.C.W.); yonemaru.junichi334@naro.go.jp (J.-i.Y.); 2 Graduate School of Horticulture, Research Center for Space Agriculture and Horticulture, Chiba University, Kashiwa-no-ha 6-2-1, Kashiwa 277-0882, Chiba, Japan; anakano@chiba-u.jp; 3 Research Center for Advanced Analysis, National Agriculture and Food Research Organization, 3-1-3 Kannondai, Tsukuba 305-8604, Ibaraki, Japan; baba.koji442@naro.go.jp (K.B.); furuya.manami932@naro.go.jp (M.F.); 4 Institute of Agrobiological Sciences, National Agriculture and Food Research Organization (NARO), 3-1-3 Kannondai, Tsukuba 305-8604, Ibaraki, Japan; wakasa.yuhya442@naro.go.jp

**Keywords:** silicon, *Oryza sativa*, plant-made pharmaceuticals, hydroponics, plant factory

## Abstract

The production of plant-made pharmaceuticals (PMPs) using transgenic rice in controlled environments requires high protein yields to offset operational costs. Unlike other crops, rice has a high demand for silicon (Si). While rice accumulates Si, practical and stable methods for Si supplementation in hydroponic systems are limited by pH instability and nutrient imbalances. In this study, we developed a simple, passive strategy to supply Si by submerging silica-hydrogel fertilizer (SiHF) in the nutrient reservoir. Compared with traditional silicon sources, this approach does not exacerbate pH instability and minimizes the risk of nutrient imbalances. We evaluated its effects on plant growth, yield, phenology, and protein productivity in hydroponically grown rice. SiHF markedly increased shoot biomass and grain yield, with the optimal SiHF concentration identified at 500 g/10 L. Although root dry weight decreased, the water uptake efficiency per unit root mass significantly increased, promoting greater biomass allocation to shoots. The heading stage was advanced by up to 4 days (e.g., from 54 to 50 days) across different concentration groups. Regarding grain quality, protein concentration decreased, likely due to the dilution effect of the substantial increase in grain yield. However, the total protein yield per plant increased 1.98-fold, without remarkably altering the protein composition profile. Ultimately, this SiHF-based method of Si supplementation optimizes biomass allocation and total protein productivity in rice while accelerating the reproductive transition, without requiring complex nutrient management. This approach offers a practical strategy for establishing a foundational baseline for future PMP production efficiency in rice-based plant factory systems.

## 1. Introduction

Recent advancements have led to the development of technologies for producing plant-made pharmaceuticals (PMPs), including vaccines and various therapeutic agents [1,2,3,4,5,6]. Among these, *Nicotiana benthamiana* [7] and strawberry [8] are used as production hosts. Research has also extended to other crops, such as rice [9,10,11,12,13], lettuce [3,14], and tomato [15]. The transgenic protein accumulated in rice plant seed remains stable for at least 1.5 years at room temperature [10], and is suitable for oral administration. These characteristics make rice an advantageous platform for PMPs compared to other plant systems.

Various strategies have been explored to maximize PMP yields in rice plants. Efforts include optimizing the lighting environment [16,17,18] and improving airflow conditions [19]. While rice with lower protein content is typically preferred for food purposes due to quality considerations [20], PMP production prioritizes maximizing the yield of the target protein per unit area and per unit time. Importantly, the accumulation level of specific recombinant PMPs is fundamentally dependent on the overall protein biosynthesis capacity of the host plant. Consequently, maximizing the total protein yield serves as a crucial baseline indicator for evaluating and improving the efficacy of a PMP production platform. Controlled-environment agriculture (CEA) has strong potential to enhance protein productivity; for example, a recent study on soybeans found that vertical farming yields approximately eight times more protein per square meter than traditional open-field cultivation [21]. Additionally, plant factory environments significantly enhance metabolic diversity and accumulation of valuable compounds in rice [22], supporting the use of such systems for high-value pharmaceutical production.

Despite these promising approaches, effective management strategies to further increase protein productivity in rice, particularly under controlled-environment agriculture, remain limited [23]. Unlike other crops commonly grown in plant factories, such as lettuce, rice has a particularly high demand for silicon. The benefits of applying Silicon (Si) to soil and paddy fields have been well documented, noting improvements in rice yield [24,25]. However, current methods for enriching silicon in hydroponic nutrient solutions are not well established. Importantly, hydroponic systems in plant factories are highly sensitive to fluctuations in pH and nutrient balance; because these systems often recirculate nutrient solutions in a closed environment, even minor ionic imbalances can rapidly impair plant growth. In this highly sensitive scenario, traditional Si sources, such as sodium silicate [26], potassium silicate [27], or calcium silicate [24], pose significant challenges by inducing severe pH fluctuations (alkalinization) and causing nutrient imbalances due to excessive potassium or sodium accumulation. Although sodium-free silicon sources have been tested experimentally [28], stable and practical methods for delivering Si to hydroponic nutrient solutions without altering their essential properties are required. The absence of these systems is a critical bottleneck to the application of Si in hydroponic and plant factory-based rice cultivation.

To overcome these challenges, silica-hydrogel fertilizer (SiHF) presents a promising alternative. SiHF is a highly pure, slow-release silica material that gradually supplies plant-available Si into water without releasing unnecessary counterions, such as sodium or potassium, that could disrupt the established nutrient balance [29]. While silica hydrogel materials have been previously proven to enhance plant growth and crop yield in agriculture, particularly in rice cultivation [30], their application as a stable Si source in closed hydroponic systems remains unexplored. Based on these material properties, we selected SiHF as an ideal candidate. Given the crucial role of Si in rice growth, the primary objectives of this study were to determine the optimal SiHF concentration, elucidate the underlying physiological mechanisms of Si uptake and biomass allocation, and verify the overall practicality of this method in general plant factory systems. We hypothesized that SiHF could steadily supply Si without requiring continuous pH regulation or causing nutrient imbalances. Ultimately, this approach aims to optimize biomass allocation and enhance the overall efficiency and productivity of PMPs in hydroponically grown rice, thereby substantially reducing operational costs associated with complex nutrient management and promoting the economic viability and commercialization of PMP production systems.

## 2. Results

### 2.1. Silicon Supply Method Using Silica Hydrogel Fertilizer

**Figure 1:**
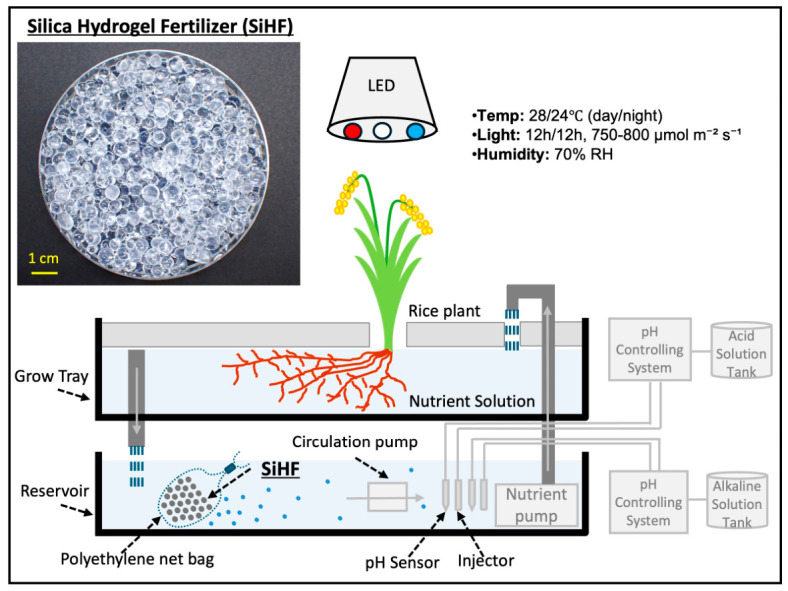
Schematic diagram of the hydroponic system incorporating the simple silica application method. Silica hydrogel fertilizer (SiHF) beads (inset photograph, scale bar = 1 cm) are enclosed in a polyethylene net bag and submerged in the reservoir. The eluted silicic acid is circulated throughout the system by the nutrient pump, maintaining a continuous supply to the rice plants without requiring complex management.

Silica hydrogel fertilizer (SiHF) was used as the silicon source, placed in a polyethylene net bag, and positioned within a reservoir (Figure 1). The silicic acid eluted into the nutrient solution was circulated via a nutrient pump, ensuring continuous replacement of the solution in the grow tray.

### 2.2. Yield and Quality Assessment

**Figure 2:**
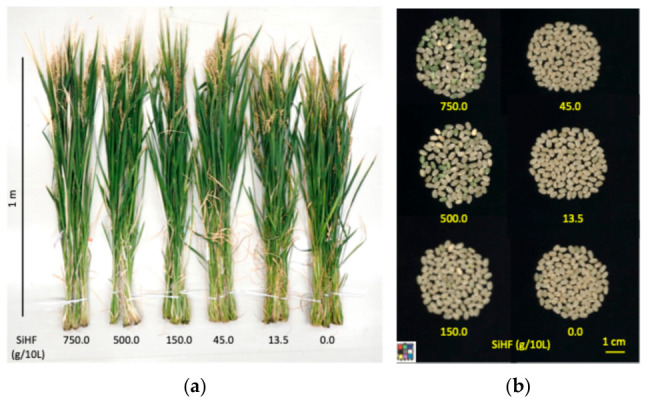
Harvested rice plants and grains. (**a**) Three plants grown under 0, 13.5 g, 45 g, 150 g, 500 g, and 750 g/10 L silica hydrogel fertilizer (SiHF); (**b**) brown rice harvested under each growth condition.

**Figure 3:**
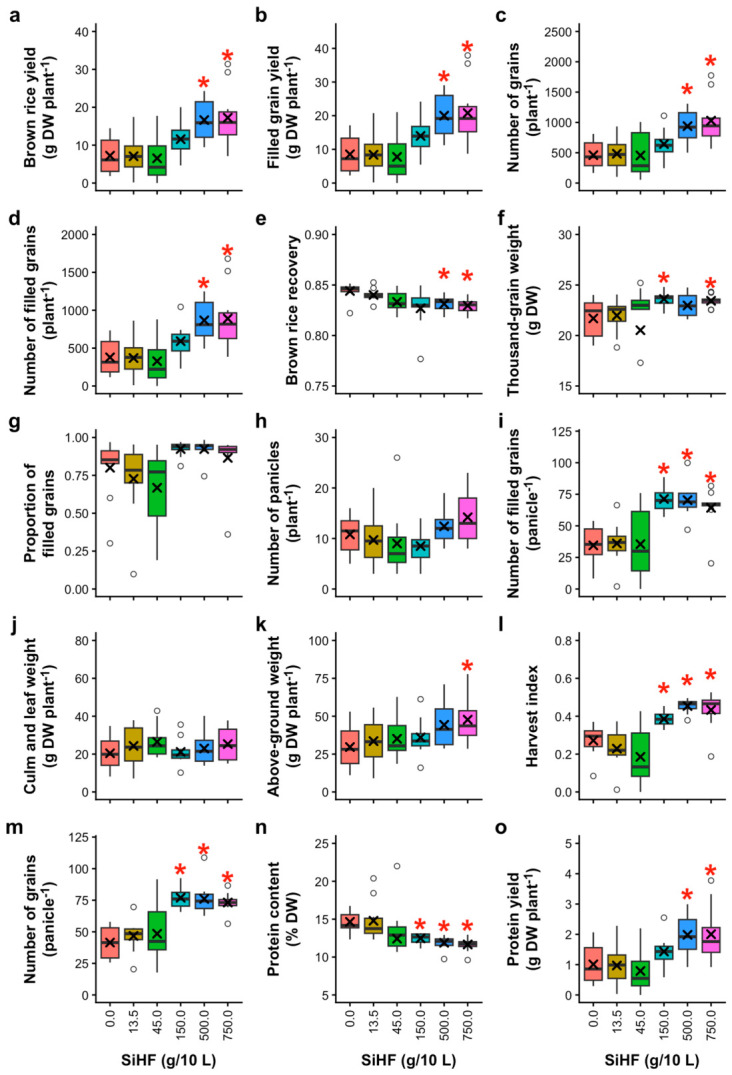
Effects of silica hydrogel fertilizer (SiHF) application rates on rice yield, yield components, biomass, and grain quality. (**a**–**o**) Agronomic traits evaluated across six SiHF treatments ranging from 0.0 to 750.0 g/10 L: (**a**) brown rice yield, (**b**) filled grain yield, (**c**) number of grains per plant, (**d**) number of filled grains per plant, (**e**) brown rice recovery, (**f**) thousand-grain weight, (**g**) proportion of filled grains, (**h**) number of panicles per plant, (**i**) number of filled grains per panicle, (**j**) culm and leaf weight, (**k**) above-ground weight, (**l**) harvest index, (**m**) number of grains per panicle, (**n**) protein content, and (**o**) protein yield. In the box plots, the horizontal line represents the median, the box spans the interquartile range (IQR), and the whiskers extend to the furthest data points within 1.5 × IQR. Open circles indicate outliers, and the black cross mark (×) within each box denotes the mean value of the biological replicates (*n* = 9–10). Asterisks (* *p* < 0.05) indicate statistically significant differences compared with the control (0.0 g/10 L). For these comparisons, significance was determined using Dunnett’s test; where the assumption of homogeneity of variance was violated (assessed via Bartlett’s test), a heteroskedasticity-consistent robust covariance matrix was applied to prevent Type I errors. The raw dataset corresponding to these traits is provided in Data S1.

The application of SiHF had pronounced effects on rice plant yield and growth parameters (Figure 2 and Figure 3). Brown rice yield and filled-grain yield per plant, as well as total and filled-grain number per plant, demonstrated clear dose-dependent increases with rising SiHF concentrations. Notably, these yields were significantly higher at SiHF levels of 500.0 and 750.0 g/10 L than in the control (0.0 g/10 L).

**Figure 4:**
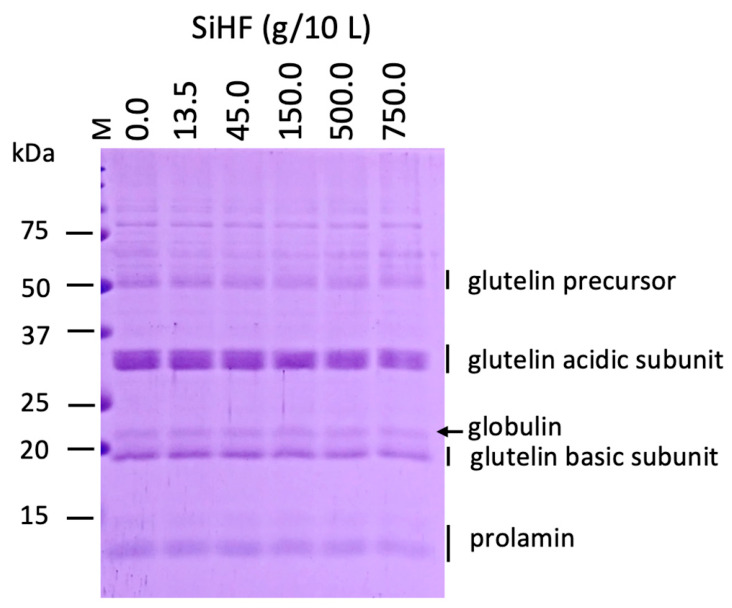
SDS-PAGE analysis of total proteins from brown rice cultivated under different silica hydrogel fertilizer (SiHF) conditions. Total proteins were extracted from brown rice and separated by 12% SDS-PAGE, with samples loaded based on equal seed weight, followed by staining with Coomassie Brilliant Blue. Lane M: Molecular weight marker (sizes are indicated in kDa on the left). Lanes 1–6: Samples from brown rice cultivated under 0.0, 13.5, 45.0, 150.0, 500.0, and 750.0 g/10 L SiHF conditions, respectively.

The observed increase in total grain number was primarily driven by a substantial increase in the number of grains per panicle, rather than changes in the number of panicles per plant, which did not differ significantly among treatments. Furthermore, the thousand-grain weight (assessed for filled brown rice only) significantly increased at the 150.0 and 500.0 g/10 L SiHF application rates compared with the control. Conversely, the brown rice recovery rate decreased significantly at higher SiHF application rates (500.0 g/10 L and above). The proportion of filled grain, however, showed no significant differences relative to the control group across all SiHF treatments. Regarding biomass, above-ground dry weight was notably higher at 750.0 g/10 L, than in the control. In terms of grain quality, the grain protein content decreased significantly from 14.6% in the control to 12.5%, 11.9%, and 11.7% at SiHF application rates of 150.0, 500.0, and 750.0 g/10 L, respectively. However, driven by the substantial increase in overall grain production, the total protein yield per plant ultimately increased, reaching 1.98 and 2.00 g DW at 500.0 and 750.0 g/10 L, respectively, compared with 1.00 g DW in the control. SDS-PAGE analysis shows that the gross banding pattern was not markedly altered (Figure 4). The complete raw dataset detailing the yield components, plant biomass, and protein content across all treatments is available in Data S1.

### 2.3. Analysis of the Relative Contribution to Protein Yield

**Figure 5:**
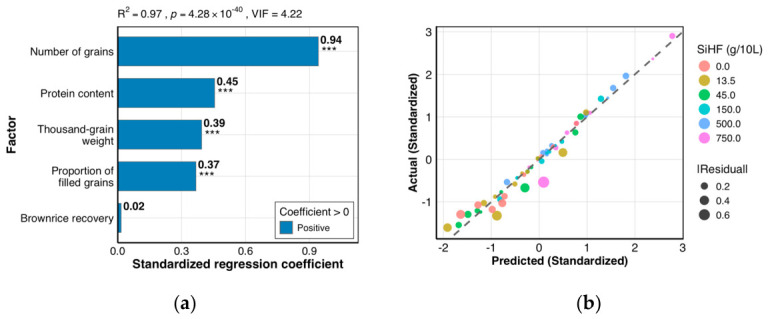
Multiple regression analysis of total protein yield per plant. (**a**) Standardized regression coefficients (β) for five explanatory variables—number of grains, seed protein content (% DW), thousand-grain weight (g DW, filled brown rice only), proportion of filled grains, and brown rice recovery. All variables were *z*-standardized prior to modeling. Model fit (top): *R*^2^ = 0.97; overall F-test, *p* < 0.001; maximum variance inflation factor (VIFmax) = 4.22. Bars are ordered by |β|; *** *p* < 0.001; *n* = 60 plants (10 replicates per SiHF application rate, six levels). (**b**) Comparison between the observed and predicted total protein yield per plant. The dashed line represents the 1:1 line (y = x) of perfect agreement.

A multiple regression analysis was conducted to evaluate the relative contribution of the predefined yield components to the total protein yield. Because total protein yield is derived from these specific components, the model inherently exhibited a high coefficient of determination (R^2^ = 0.97, *p* < 0.001). The variance inflation factor (VIF) of 4.22 indicated no substantial multicollinearity among the variables, ensuring the reliable interpretation of each component’s weight. Standardized regression coefficients were utilized to determine the relative importance of each factor (Figure 5a). Grain number emerged as the most dominant contributor, exhibiting the largest standardized positive coefficient (0.94, *p* < 0.001). Protein content (0.45, *p* < 0.001), thousand-grain weight (0.39, *p* < 0.001), and filled grain ratio (0.37, *p* < 0.001) also contributed significantly. Conversely, brown rice recovery exhibited a negligible relative effect. Furthermore, the comparison between predicted and observed values (Figure 5b) visually confirmed this expected mathematical alignment across all experimental treatments, rather than serving as an independent validation of predictive accuracy.

### 2.4. Effect of SiHF Application on Plant Growth Characteristics

**Figure 6:**
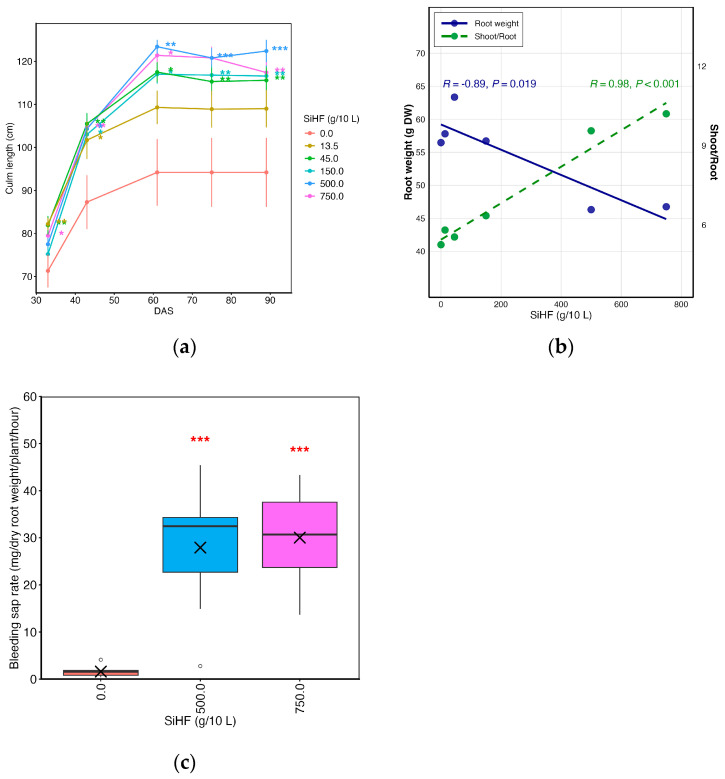
Effects of silica hydrogel fertilizer (SiHF) application on shoot elongation and root characteristics of rice plants. (**a**) Time course changes in culm length from 33 to 89 days after sowing (DAS). (**b**) Dose–response relationship between SiHF application rate and plant biomass allocation. Correlation between SiHF application rate and root dry weight (left axis, g DW) and shoot-to-root ratio (right axis) at harvest. Shoot dry weight includes panicles. Solid blue line: linear regression model for root dry weight (*r* = −0.89, *R^2^* = 0.79, *p* = 0.019), indicating a significant reduction in root biomass with increasing SiHF. Dashed green line: linear regression model for the shoot-to-root ratio (*r* = 0.98, *R^2^* = 0.96, *p* < 0.001), demonstrating a strong shift toward shoot-prioritized biomass allocation induced by SiHF. (**c**) Bleeding sap rate per unit root dry weight measured at harvest, indicating root pressure-driven xylem transport. Data in (**a**) are presented as means ± standard error (SE) (*n* = 5). In the box plot (**c**), the center line represents the median, the box limits indicate the 25th and 75th percentiles, whiskers extend to the minimum and maximum values, and “×” indicates the mean value. Asterisks (* *p* < 0.05, ** *p* < 0.01, *** *p* < 0.001) indicate statistically significant differences compared with the control (0.0 g/10 L) in panels (**a**,**c**). For these comparisons, significance was determined using Dunnett’s test; where the assumption of homogeneity of variance was violated (assessed via Bartlett’s test), a heteroskedasticity-consistent robust covariance matrix was applied to prevent Type I errors.

The application of SiHF significantly affected the morphological characteristics of rice plants. Notably, culm length increased markedly in the SiHF-treated plots compared to the control, with this effect becoming more pronounced during the later stages of growth (Figure 6a). This suggests that Si supplementation enhances vertical growth under hydroponic conditions. Conversely, a strong and significant negative correlation was observed between the SiHF application rate and root weight (R = −0.89, *p* = 0.019; Figure 6b). As the SiHF application rate increased, the final root dry weight significantly decreased. Despite this reduction in root biomass, the root pressure-driven water uptake efficiency remained high. In particular, bleeding sap volume—an indicator of root pressure and water uptake efficiency—was significantly higher in plants treated with 500.0 or 750.0 g/10 L SiHF than in controls (*p* < 0.001; Figure 6c). Collectively, these results indicate that SiHF application prompts a reallocation of biomass from roots to shoots including panicles while enhancing the physiological efficiency of the remaining root system.

### 2.5. Effect of SiHF on Phenology and Silicon Uptake Dynamics

**Figure 7:**
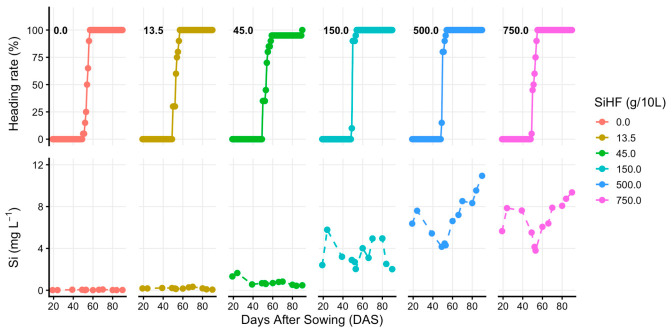
Temporal changes in heading rate and silicon (Si) concentration in the nutrient solution under different silica hydrogel fertilizer (SiHF) regimes. (**Upper row**) Cumulative heading rate (%) from 20 to 90 days after sowing (DAS). The sigmoidal curves for high SiHF application rates (150.0–750.0 g/10 L) shift to the left compared to the control (0.0 g/10 L), indicating an acceleration of the heading event. (**Lower row**) Monitoring of Si concentration (mg L^−1^) in the nutrient solution. Data points represent the measured Si concentration at each sampling time. In high SiHF treatments, a distinct fluctuation pattern was observed—decreasing around 50 DAS (corresponding to the heading stage) and subsequently increasing—indicating active Si consumption by the plants during the reproductive phase.

The SiHF application also significantly affected the phenological development of rice plants by accelerating the heading stage. The heading date, determined when 50% of the plants (*n* = 10) had reached heading, was recorded as 54 days after sowing (DAS) for the control (0.0 g/10 L) and 45.0 g/10 L treatment groups (Figure 7). Similarly, the 13.5 g/10 L treatment reached heading at 53 DAS. In contrast, higher concentrations of SiHF resulted in earlier transitions to the reproductive phase: the 150.0 and 500.0 g/10 L treatments achieved heading at 50 DAS, and the 750.0 g/10 L treatment at 51 DAS. These observations demonstrate that sufficient Si availability shortens the vegetative growth period, advancing heading by approximately 3–4 days compared with Si-deficient (control) conditions.

**Figure 8:**
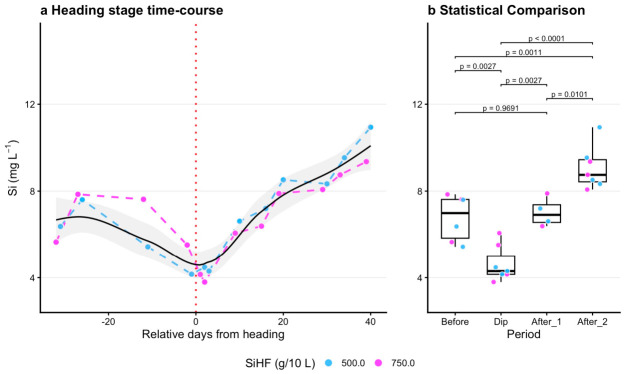
Dynamics of silicon (Si) concentration in the nutrient solution relative to the heading event. (**a**) Time course of Si concentration (mg L^−1^) in the nutrient solution aligned with the heading date (=0); solid black line: moving average, shaded area: confidence interval, dashed lines: trends for 500.0 and 750.0 g/10 L SiHF treatments. A specific decrease (dip) in Si concentration coincides with the heading event. (**b**) Statistical comparison of Si concentrations across four phases defined by relative days from heading (*d*): Before (*d* ≤ −10), Dip (−10 < *d* < 10), After 1 (10 ≤ *d* < 20), and After 2 (*d* ≥ 20). Box plots show the median (center line), interquartile range (box), and data points (dots). Statistical significance among the stages was determined using Tukey’s honestly significant difference (HSD) test, following the confirmation of homogeneity of variance via Bartlett’s test.

Additionally, monitoring Si concentrations in the nutrient solution revealed uptake patterns closely associated with phenological changes (Figure 7 and Figure 8). High SiHF treatments were characterized by dynamic fluctuations in reservoir Si concentration. Detailed analysis related to the heading date identified a distinct decline (dip) in Si concentration precisely at the time of heading (0 days relative to heading). Statistical analysis confirmed that Si levels during heading were significantly lower than in the periods before and after heading (*p* < 0.01), indicating temporally specific Si uptake aligned with the onset of the reproductive stage. The comprehensive time-course dataset for phenological development and silicon concentration is provided in Data S2.

**Figure 9:**
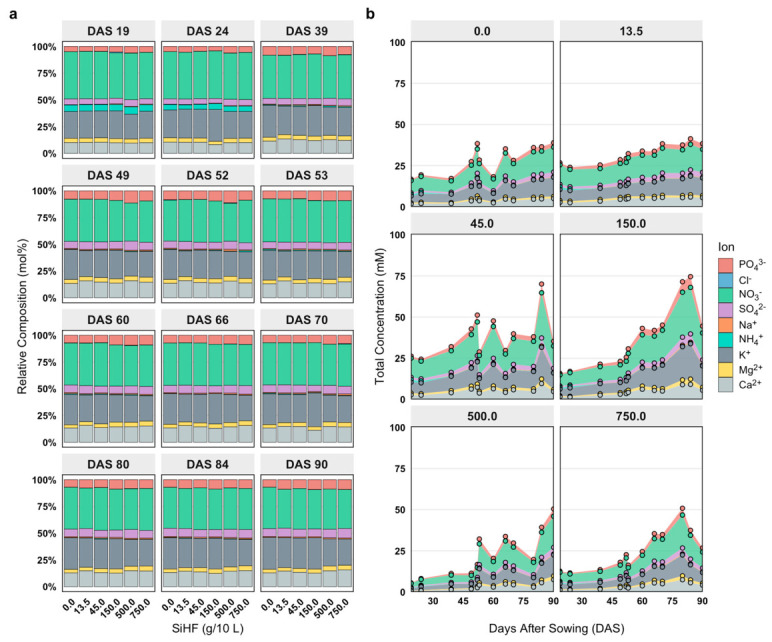
Relative molar composition and temporal dynamics of absolute ion concentrations under different silica hydrogel fertilizer (SiHF) treatments. (**a**) Cross-sectional comparison of the relative composition (mol%) of major anions (chloride, nitrate, phosphate, and sulfate) and cations (ammonium, calcium, magnesium, potassium, and sodium) among different SiHF application rates, ranging from 0.0 to 750.0 g/10 L, at each sampling growth stage from 19 to 90 days after sowing (DAS). The 100% stacked bar charts illustrate the robust maintenance of the nutrient balance. Simple linear regression analysis followed by the Benjamini–Hochberg (BH) false discovery rate correction revealed no significant linear trends in any individual ion proportions across the SiHF application rates at all observed growth stages. (**b**) Time-course changes in the absolute concentration (mM) of the nine measured ions. Stacked area charts illustrate the temporal dynamics of nutrient accumulation across varying SiHF application rates, with total molar concentrations plotted at each sampling point (indicated by open circles). The relative molar composition in (**a**) was calculated based on the absolute millimolar (mM) concentrations shown in (**b**). Detailed statistical outputs for the composition analysis, including adjusted values for each ion, are provided in Data S3. Comprehensive absolute concentration datasets are provided in Data S4.

The relative molar composition (mol%) of the nine inorganic ions (chloride, nitrate, phosphate, sulfate, sodium, ammonium, potassium, magnesium, and calcium) in the nutrient solution remained highly stable across all SiHF application rates throughout the entire cultivation period. Statistical analysis revealed no significant linear trends in the proportion of any individual ion in response to SiHF treatments at any growth stage, demonstrating the robust nutrient balance maintained within this system (Figure 9, Data S3). The time-course dataset including both absolute concentrations (in mg/L and mM) and relative molar compositions is available in Data S4.

## 3. Discussion

### 3.1. Optimization of Biomass Allocation and Efficiency of Root Function by SiHF

A key outcome of this study is the observation that SiHF application markedly influenced plant morphogenesis. Notably, while shoot biomass and grain yield were significantly increased, root dry weight decreased considerably. Conventionally, a reduction in root mass under soil-cultivation conditions is often interpreted as indicating impaired nutrient uptake or an increased risk of lodging. However, in a controlled hydroponic environment, this phenomenon can be interpreted as evidence of the plant’s “optimization of biomass allocation.” Under nutrient-limited or stress conditions, plants prioritize allocating resources to roots to expand their foraging surface area [31]. In the current study, the continuous, passive supply of available silicic acid via SiHF likely reduced the metabolic burden associated with root system maintenance, enabling the allocation of more energy and carbon skeletons to above-ground growth, particularly reproductive organs (grains). This reallocation is substantiated by the significant increase in the shoot-to-root (S/R) ratio. Additionally, the significantly higher bleeding sap rate per unit root weight in high-SiHF treatments suggests that, despite reduced root mass, water uptake efficiency per root unit was substantially improved.

Sakaigaichi et al. [32] reported that bleeding rates typically peak at the heading stage and then decline due to root senescence. In contrast, our findings suggest that this root function remained at a high level even at the harvest stage (90 DAS) due to SiHF application. Previous research has established that Si plays a vital role in mitigating oxidative stress by enhancing antioxidant enzyme activities [33]. The sustained root function likely contributed to improved shoot performance. San-oh et al. [34] demonstrated that cytokinin flux via xylem sap positively correlates with the maintenance of leaf photosynthetic rate and Rubisco content during ripening. Accordingly, the SiHF-induced functional maintenance of the root system likely maintained sufficient cytokinin levels, thereby delaying senescence and maximizing protein yield. Similarly, in tomato, bleeding rate positively correlates with fruit fresh weight [35]. This supports the hypothesis that enhanced root pressure contributes to sink organ development. Ma and Yamaji [36] reported that rice features an efficient uptake system mediated by Lsi transporters; the current results suggest that SiHF enhances this system, promoting the optimization of the root system toward lower biomass and higher water uptake efficiency, potentially contributing to yield enhancement. However, because root samples in this study were pooled and weighed per cultivation condition, definitive evaluations of individual root morphological efficiency could not be performed. Fully elucidating the transition to a morphologically highly efficient root system will require further detailed root architectural studies.

### 3.2. Specific Silicon Requirements During the Reproductive Phase and Its Role in Growth Cycle Acceleration

Nutrient solution monitoring revealed a sharp decrease in Si concentration during the heading stage. This observation physiologically suggests a distinct and rapid requirement for Si by rice plants during their transition to the reproductive phase. Ma et al. [37] established that approximately 65% of total Si uptake in rice occurs during the reproductive stage. A substantial portion of absorbed Si is localized in the husks (lemma and palea), contributing to mechanical stability and resistance to biotic and abiotic stresses [38]. The detected “dip” in Si concentration likely reflects the active translocation of Si to floral organs, facilitating silicification. Furthermore, it is worth considering the potentially distinct silicon uptake dynamics between field and hydroponic environments. While field-grown rice also exhibits peak Si uptake during the reproductive stage [37], the massive buffering capacity of the soil matrix may obscure acute temporal fluctuations in available Si. Conversely, in our closed hydroponic system, a highly synchronized, sharp “dip” in Si concentration was observed precisely at heading. This suggests a rapid, concentrated Si demand for panicle development and silicification, a phenomenon that might be more readily detectable in the limited volume of a hydroponic reservoir. However, because plant tissue Si content was not directly quantified in this study, further investigations are necessary to definitively attribute this solution Si decrease solely to plant uptake.

Additionally, treatments with high SiHF levels (150–750 g/10 L) advanced the heading date by 3–4 days compared with the control, suggesting that sufficient Si availability not only strengthens the physical structure but also expedites progression into the reproductive stage. Regarding the acceleration of heading, the molecular mechanisms remain to be fully elucidated. As a future research direction, we propose investigating the crosstalk between Si-enhanced carbon metabolism and floral transition pathways. It is widely recognized that excessive nitrogen supply prolongs vegetative growth and delays heading in rice [39,40]. In our highly nitrogen-rich hydroponic environment, however, the massive influx of photosynthates facilitated by Si application may rapidly increase the internal carbon-to-nitrogen (C/N) ratio. This shift could act as an early endogenous signal to upregulate florigen genes, such as Hd3a and RFT1 [39]. Additionally, the role of Si in modulating phytohormone dynamics warrants transcriptomic and metabolomic profiling to decipher how Si mitigates developmental bottlenecks and triggers an earlier reproductive transition.

In the context of PMP production in plant factories, where annual productivity depends on the number of cultivation cycles, this potential acceleration of the reproductive cycle represents a considerable advantage in terms of operational costs and efficiency. Although all plants in this study were uniformly harvested at 90 days after sowing (DAS), this physiological shift presents substantial potential for PMP production in plant factories. The advanced heading could potentially allow for an earlier harvest, thereby shortening the overall production cycle. Alternatively, maintaining a fixed cultivation period would extend the grain-filling duration, contributing to further yield optimization. Future studies should precisely evaluate maturity and harvest readiness to verify the extent to which SiHF application can increase the number of annual cultivation cycles and improve operational efficiency.

### 3.3. Protein Content and Yield Trade-Off in PMP Production

For PMP production, the total recovery of the target protein per unit area and time remains the principal performance metric. Our observations revealed an inverse relationship between seed protein concentration (% DW) and increasing SiHF levels. This phenomenon is widely recognized in cereal crops as the “dilution effect” [41], in which carbohydrate accumulation during grain filling surpasses nitrogen accumulation [42]. A conceptually similar dilution dynamic is also critical in medicinal plant production; for instance, the relative concentrations of pharmaceutical ingredients, such as polyphenol and ephedrine-type alkaloids in Ephedra sinica, significantly decrease when massive seasonal carbohydrate accumulation drives structural tissue development [43]. Although we did not directly quantify the grain starch content—which represents a limitation of the current study in fully elucidating this dynamic—the significant increases in thousand-grain weight and filled-grain yield at higher SiHF applications strongly support this hypothesis. Silicon deposition in leaf tissues is known to maintain erect leaf posture, thereby improving light interception and photosynthetic activity [44]. We postulate that this enhanced photosynthetic capacity accelerated the massive influx of carbohydrates (primarily starch) into the grain sink in our study. Consequently, while the absolute protein yield per plant increases, the relative protein concentration is diluted by this disproportionately large carbohydrate mass.

SDS-PAGE analysis further showed that the gross banding pattern was not markedly altered among treatments, and the bands corresponding to major seed proteins, as estimated from their molecular weights, did not show conspicuous changes (Figure 4). These observations suggest that high SiHF treatments did not induce major metabolic shifts or stress responses that impair protein synthesis, at least at the level detectable by SDS-PAGE. Instead, the decrease in protein concentration reflects a general dilution due to increased carbohydrate production (Figure 3n). Importantly, the marked increase in grain yield observed in SiHF-treated groups compensated for the lower protein concentration, resulting in a significant increase in total protein yield per plant. Future studies will be required to evaluate protein composition in more detail, including target proteins as well as their precursors and related proteins involved in their metabolism.

The contribution analysis based on multiple regression revealed that grain number made the largest relative contribution to the increase in total protein yield (Figure 5a). Together with the observed effects of SiHF on biomass allocation (Figure 3), these results suggest that SiHF indirectly increases total protein yield primarily through an increase in grain number. These outcomes support an approach focused on maximizing biomass (specifically grain number), even at the expense of a slight dilution of protein concentration, as a rational strategy for establishing a robust baseline platform for future PMP production.

### 3.4. Optimization and Scalability for Industrial Application

Traditionally, hydroponic Si supplementation has relied on potassium silicate or sodium silicate; however, these compounds often cause instability and precipitation, which can block irrigation systems and necessitate more complex management protocols [45]. The approach established in this study employs SiHF and is notably simple—requiring only the placement of a net bag in the reservoir—while facilitating a passive supply of Si that aligns with plant requirements, without disrupting pH balance or solution composition. This combination of operational simplicity and enhanced productivity reduces the technical barrier to implementation, positioning the method as an attractive solution for advancing the industrial-scale adoption of foundational platforms for future PMP production within large-scale plant factories.

Given that the protein yield showed comparable performance between the 500 g/10 L and 750 g/10 L treatments, we recommend an optimal SiHF application concentration of 500 g/10 L to balance yield and cost-effectiveness. Regarding the use cost per unit area, although the market price of SiHF must be factored in, this method offers substantial economic competitiveness compared to traditional silicon sources. Specifically, because SiHF minimizes the leaching of undesirable cations such as sodium, it maintains solution integrity and eliminates the need for frequent nutrient solution replacements, thereby reducing waste liquid treatment costs and extending the operational replacement cycle. Furthermore, its passive diffusion mechanism simplifies the required control systems while ensuring nutrient uniformity. Crucially, this optimized approach achieved a twofold increase in seed protein yield. Given the exceptionally high added value of PMPs, such a substantial yield enhancement is more than sufficient to fully offset the operational and material costs of SiHF, confirming its commercial viability for industrial-scale adoption.

However, several limitations of the current study should be acknowledged. First, our physiological evaluation was strictly limited to a single rice cultivar, “Kitaake,” which is utilized as a parental line in PMP production research [9]. The accumulation efficiency and morphological responses to SiHF may vary among different agronomic or transgenic rice cultivars with diverse genetic backgrounds. Second, this investigation focused on a single growth cycle up to 90 DAS; thus, the long-term effects of chronic SiHF utilization—such as the structural stability of the hydrogel over multi-cycle operations—and its applicability under varying environmental conditions, including different nutrient temperature, light qualities, or alternative nutrient composition, remain to be fully evaluated. Future research incorporating multi-cultivar trials and long-term validation across diverse closed-environment setups will be essential to establish the universal scalability of this baseline platform.

## 4. Materials and Methods

### 4.1. Plant Materials and Seedling Preparation

Rice (*Oryza sativa* L. cv. Kitaake) seeds were surface-sterilized by immersion in 70% ethanol (Maiple Co., Ltd., Osaka, Japan), followed by treatment with a 0.25% sodium hypochlorite solution (FUJIFILM Wako Pure Chemical Corporation, Osaka, Japan). Fifty sterilized seeds were placed on 90-mm Petri dishes containing ion-exchanged water and incubated for 3 days at 30 °C in the dark, followed by 2 days under continuous light (approximately PPFD 120 µmol m^−2^ s^−1^) at 30 °C (FCI-280GEC, AS ONE Corporation, Osaka, Japan). Germinated seedlings were transferred to polyurethane foam blocks (25 mm × 25 mm × 25 mm) for hydroponic cultivation.

During the nursery stage, the nutrient solution comprised 0.25 units Otsuka A formula (electrical conductivity [EC] 0.65 dS m^−1^, pH 5.0–6.0; OAT Agrio, Tokyo, Japan) during the first week, followed by 0.5 units Otsuka A formula (EC 1.3 dS m^−1^). OAT HOUSE No.5 (OAT Agrio, Tokyo, Japan) was supplemented weekly at 3 mg L^−1^. After two weeks of seedling establishment, plants were grown in an incubator (Nippon Medical & Chemical Instruments Co., Ltd., Osaka, Japan) under a 22 h photoperiod at 28 °C/24 °C (day/night), PPFD 685 µmol m^−2^ s^−1^, and CO_2_ concentration of 1500 ppm.

Upon completion of the nursery stage, seedlings were transplanted into a hydroponic system (Home Hyponica Sarah+, KYOWA Co., Ltd., Osaka, Japan) equipped with pH Dosing System (HI 981412, Hanna Instruments Japan Co., Ltd., Chiba, Japan) to manage pH fluctuations caused mainly by plant root exudates. The plants were then cultivated to maturity in an agro-environment emulator (Espec Mic Co., Ltd., Aichi, Japan) under a 12 h photoperiod at 28 °C/24 °C (day/night), PPFD 750–800 µmol m^−2^ s^−1^, and CO_2_ concentration of 1000 ppm. During this cultivation stage, OAT HOUSE No.5 (OAT Agrio, Tokyo, Japan) was applied every 30 days at 30 mg L^−1^.

To evaluate the applicability of SiHF to hydroponic systems, Water Silica (Katakura & Co-op Agri Corporation, Tokyo, Japan) beads were placed in polyethylene net bags and submerged in a nutrient reservoir. The SiHF product is specified to contain 17.0% soluble silicate. The bead diameter was approximately 4–7 mm. SiHF loading per 10 L of reservoir volume was set to 0, 13.5, 45, 150, 500, or 750 g, representing the initial solid mass introduced (not the dissolved Si concentration) to evaluate dose-dependent effects under practical hydroponic conditions. Due to spatial and logistical constraints, a single continuous nutrient reservoir and cultivation tray were established for each SiHF treatment. Within each tray, 20 individual plants were cultivated. The pH and electrical conductivity (EC) of the nutrient solutions were monitored continuously (Data S5), and the pH was maintained at approximately 5.0–6.0. On days when the EC or pH required manual adjustment, measurements were taken twice: immediately before and after the adjustment. Water was added to the nutrient solution every 2 to 3 days to restore the original water level lost through evaporation and transpiration.

Plants were harvested 90 DAS. At harvest, 10 out of the 20 plants in each tray were sampled for evaluation of agronomic traits. At harvest, panicles were isolated and dried in a desiccator containing silica gel for two weeks, while the remaining plant parts were oven-dried at 60 °C for 5 days and weighed. Roots were pooled and weighed for each cultivation condition after oven drying.

### 4.2. Measurement of Bleeding Sap

At harvest (90 DAS), plant shoots were excised approximately 10 cm above the root base. A pre-weighed cotton pad was placed on the cut stump and secured with plastic wrap to minimize evaporation. After 1 h of collection, the cotton pad was reweighed to determine the amount of bleeding sap. The bleeding sap rate was calculated as the weight of sap exuded per unit root dry weight (mg g^−1^ root DW).

### 4.3. Silicon Analysis

Nutrient solution samples collected from the reservoir were immediately centrifuged at 13,420× *g* for 10 min to remove suspended solids. The supernatant was transferred to polypropylene tubes and stored at 4 °C until analysis. Samples were not acidified prior to analysis to maintain the integrity of dissolved silicon species, as silicic acid is known to undergo polymerization and precipitation under acidic conditions, leading to the loss of soluble silicon; this approach is consistent with established analytical guidelines [46]. Calibration curves were constructed by stepwise dilution of a certified Si standard solution (Si 1000; Kanto Chemical Co., Inc., Tokyo, Japan; JCSS grade for chemical analysis, atomic absorption spectrometry, and ICP spectrometry) with Milli-Q ultrapure water (Millipore, Burlington, MA, USA). All standard solutions were prepared in advance and used within three days to maintain stability. Silicon concentrations were determined using an Agilent 5800 VDV ICP-OES (Agilent Technologies, Santa Clara, CA, USA) at an emission wavelength of 250.690 nm. The limit of quantitation (LOQ, 0.2 mg L^−1^) was defined as ten times the SD of Si-free nutrient solution samples (*n* = 12). ICP-OES operating conditions are provided in Tables S1 and S2.

### 4.4. Anion and Cation Analysis of the Nutrient Solution

The concentrations of anions (chloride, nitrate, phosphate, and sulfate) and cations (sodium, ammonium, potassium, magnesium, and calcium) in the hydroponic nutrient solution were determined using an ion chromatography (IC) system (Shimadzu Corporation, Kyoto, Japan) operated in non-suppression mode. Before sampling, water was added to the nutrient solution to restore the original water level lost through evapotranspiration. Prior to analysis, the collected nutrient solution samples were diluted 10-fold with ultrapure water and filtered through a 0.45 µm hydrophilic polypropylene membrane filter (Shimadzu GLC Ltd., Tokyo, Japan) to remove particulate matter. For anion analysis, chromatographic separation was achieved using a Shim-pack IC-A3 analytical column (150 mm × 4.6 mm i.d., Shimadzu) equipped with a Shim-pack IC-GA3 guard column (10 mm × 4.6 mm i.d., Shimadzu) maintained at 35 °C. The mobile phase consisted of 8 mM *p*-hydroxybenzoic acid, 3.2 mM Bis-Tris, and 50 mM boric acid, delivered at an isocratic flow rate of 1.2 mL min^−1^. For cation analysis, separation was performed using a Shim-pack IC-C4 analytical column (150 mm × 4.6 mm i.d., Shimadzu) equipped with a Shim-pack IC-GC4 guard column (8 mm × 3 mm i.d., Shimadzu) maintained at 35 °C. The mobile phase consisted of 2.5 mM oxalic acid at an isocratic flow rate of 1.0 mL min^−1^. In both analytical modes, the eluted ions were monitored using a conductivity detector (CDD-10Avp, Shimadzu). The sample injection volume was 25 µL. Individual ions were identified by comparing their retention times with those of corresponding reference standards. Quantitative calibration curves were constructed using a series of multi-anion and multi-cation standard solutions prepared from certified standard materials (FUJIFILM Wako Pure Chemical Corporation, Osaka, Japan).

### 4.5. Total Protein Content Analysis

A total of 1.5 mL of extraction buffer (50 mM Tris-HCl, 4M urea, 4% SDS, 5% 2-mercaptoethanol) was added to 30 mg of seed powder, followed by continuous vortexing for 30 min at room temperature. The mixture was centrifuged at 17,970× *g* for 5 min at room temperature, and the supernatant containing total proteins was used for protein analysis. Total protein content was measured with an RC DC protein assay kit (Bio-Rad Laboratories, Inc., CA, USA).

### 4.6. SDS-PAGE Analysis

Seed powder (10 mg) was mixed with 2 mL of extraction buffer (50 mM Tris-HCl, 8 M urea, 4% SDS, 20% glycerol, 5% 2-mercaptoethanol, and 0.01% bromophenol blue) and vortexed at room temperature for 1 h. The mixture was then centrifuged at 12,000× *g* for 10 min at room temperature, and the supernatant containing total proteins was transferred to a new tube. A 4 μL aliquot of the total protein solution was loaded onto a 12% SDS-PAGE gel for electrophoresis.

### 4.7. Statistical Analysis

Statistical analyses were performed using R statistical software (version 4.5.1; R Core Team, Vienna, Austria). Data are presented as means ± standard error (SE) or standard deviation (SD). Prior to any multiple comparisons, the homogeneity of variance was evaluated using Bartlett’s test. To compare the control group (0.0 g/10 L) with the various SiHF treatments, Dunnett’s test was utilized. For variables that violated the assumption of homogeneity of variance, a heteroskedasticity-consistent robust covariance matrix was applied within the Dunnett’s framework to strictly prevent Type I errors. To assess differences in Si concentrations across phenological phases, Tukey’s honestly significant difference (HSD) test was applied following the confirmation of homoscedasticity. Statistical significance was defined at *p* < 0.05.

Multiple regression analysis was conducted to determine the relative contribution of yield components to total protein yield. For this analysis, explanatory variables were explicitly selected based on the definitive components constituting protein yield: number of grains, thousand-grain weight, proportion of filled grains, brown rice recovery, and protein content. The model was constructed using only the main effects; interaction terms were deliberately excluded to prevent multicollinearity and ensure the clear interpretability of each component’s contribution. The relationships between SiHF application rates and root characteristics were analyzed via linear regression models. Statistical significance was defined as *p* < 0.05.

To investigate the potential linear trends in individual ion proportions in response to SiHF application rates, simple linear regression analysis was applied to the relative molar composition (mol%) of each of the nine ions across the six treatment concentrations for each day after sowing (DAS). To control the inflation of the Type I error rate due to multiple hypothesis testing (108 independent tests in total), the obtained raw values were adjusted using the Benjamini–Hochberg (BH) procedure to calculate the false discovery rate (FDR).

## 5. Conclusions

This study introduced a simple and economical method for supplying Si to hydroponics systems by placing SiHF in a net bag within a reservoir. The primary benefit of this technique is that it passively supplies Si in response to plant demand, eliminating the need for complex pH management or adjustments to the nutrient solution composition. SiHF application improved plant morphology in factory environments, ensuring optimal biomass distribution by reducing root investment to maximize shoot and grain development. This strategy successfully enhances the “total protein yield” by substantially increasing grain yield, effectively compensating for the decrease in protein concentration. Additionally, the advanced heading expedites the reproductive transition, offering the potential to shorten the cultivation period, a critical factor in improving the annual turnover and profitability of plant factories. Ultimately, this technology offers a practical solution to realize the full potential of rice productivity in controlled environments, establishing a foundational baseline for future high-value PMP production.

## 6. Patents

A patent application based on the results of this work has been filed in Japan (Japanese Patent Application No. 2025-062699).

## Data Availability

The original contributions presented in this study are included in the article/Supplementary Materials. Further inquiries can be directed to the corresponding author.

## Supplementary Materials

The following supporting information can be downloaded at: https://www.mdpi.com/article/10.3390/plants15121775/s1, Data S1: Raw dataset of yield components, biomass, and protein content across SiHF treatments; Data S2: Time-course dataset of heading rate and silicon concentration in the nutrient solution; Data S3: Statistical outputs of simple linear regression analysis for the relative molar composition of major ions; Data S4: Time-course dataset including absolute concentrations (mg/L and mM) and relative molar compositions (mol%) of the major ions in the nutrient solution across SiHF treatments; Data S5: Time-course dataset of pH and electrical conductivity (EC) in the nutrient solution under different SiHF treatments; Table S1: ICP-OES operating conditions; Table S2: Candidate emission wavelengths for Si evaluated in this study.

## Abbreviations

The following abbreviations are used in this manuscript:

CEA	controlled environment agriculture
DAS	days after sowing
DW	dry weight
EC	electrical conductivity
PMP	plant-made pharmaceutical
PPFD	photosynthetic photon flux density
SDS-PAGE	sodium dodecyl sulfate–polyacrylamide gel electrophoresis
SiHF	silica hydrogel fertilizer
S/R	shoot-to-root ratio

## References

[1] Paul M., Ma J.K.-C. (2011). Plant-Made Pharmaceuticals: Leading Products and Production Platforms. Biotechnol. Appl. Biochem..

[2] Thomas D.R., Penney C.A., Majumder A., Walmsley A.M. (2011). Evolution of Plant-Made Pharmaceuticals. Int. J. Mol. Sci..

[3] Matsui T., Takita E., Oiwa S., Yokoyama A., Kato K., Sawada K. (2021). Lettuce-Based Production of an Oral Vaccine against Porcine Edema Disease for the Seed Lot System. Plant Biotechnol..

[4] Matsuda R., Matoba N. (2022). Biopharmaceutical Protein Production in Plant Factories. Clim. Biosph..

[5] Lee J., Lee S.-K., Park J.-S., Lee K.-R. (2023). Plant-Made Pharmaceuticals: Exploring Studies for the Production of Recombinant Protein in Plants and Assessing Challenges Ahead. Plant Biotechnol. Rep..

[6] Fukuzawa N., Matsuo K., Atsumi G., Tasaka Y., Mitsuda N. (2024). Plant-Made Pharmaceuticals. Plant Biotechnol..

[7] Mamedov T., Yuksel D., Ilgın M., Gürbüzaslan I., Gulec B., Mammadova G., Ozdarendeli A., Yetiskin H., Kaplan B., Islam Pavel S.T. (2021). Production and Characterization of Nucleocapsid and RBD Cocktail Antigens of SARS-CoV-2 in Nicotiana benthamiana Plant as a Vaccine Candidate against COVID-19. Vaccines.

[8] Yamaki S., Hachimura H., Ogawa M., Kanegae S., Sugimoto T., Amimoto A. (2020). Long-Term Follow-up Study after Administration of a Canine Interferon-α Preparation for Feline Gingivitis. J. Vet. Med. Sci..

[9] Takagi H., Hiroi T., Yang L., Tada Y., Yuki Y., Takamura K., Ishimitsu R., Kawauchi H., Kiyono H., Takaiwa F. (2005). A Rice-Based Edible Vaccine Expressing Multiple T Cell Epitopes Induces Oral Tolerance for Inhibition of Th2-Mediated IgE Responses. Proc. Natl. Acad. Sci. USA.

[10] Nochi T., Takagi H., Yuki Y., Yang L., Masumura T., Mejima M., Nakanishi U., Matsumura A., Uozumi A., Hiroi T. (2007). Rice-Based Mucosal Vaccine as a Global Strategy for Cold-Chain- and Needle-Free Vaccination. Proc. Natl. Acad. Sci. USA.

[11] Takaiwa F., Wakasa Y., Takagi H., Hiroi T. (2015). Rice Seed for Delivery of Vaccines to Gut Mucosal Immune Tissues. Plant Biotechnol. J..

[12] Kashima K., Yuki Y., Mejima M., Kurokawa S., Suzuki Y., Minakawa S., Takeyama N., Fukuyama Y., Azegami T., Tanimoto T. (2016). Good Manufacturing Practices Production of a Purification-Free Oral Cholera Vaccine Expressed in Transgenic Rice Plants. Plant Cell Rep..

[13] Yuki Y., Kurokawa S., Sugiura K., Kashima K., Maruyama S., Yamanoue T., Honma A., Mejima M., Takeyama N., Kuroda M. (2024). MucoRice-CTB Line 19A, a New Marker-Free Transgenic Rice-Based Cholera Vaccine Produced in an LED-Based Hydroponic System. Front. Plant Sci..

[14] Matsui T., Asao H., Ki M., Sawada K., Kato K. (2009). Transgenic Lettuce Producing a Candidate Protein for Vaccine against Edema Disease. Biosci. Biotechnol. Biochem..

[15] Sun H.-J., Kataoka H., Yano M., Ezura H. (2007). Genetically Stable Expression of Functional Miraculin, a New Type of Alternative Sweetener, in Transgenic Tomato Plants. Plant Biotechnol. J..

[16] Matsuda R., Ohashi-Kaneko K., Fujiwara K., Goto E., Kurata K. (2004). Photosynthetic Characteristics of Rice Leaves Grown under Red Light with or without Supplemental Blue Light. Plant Cell Physiol..

[17] Ohashi-Kaneko K., Matsuda R., Goto E., Fujiwara K., Kurata K. (2006). Growth of Rice Plants under Red Light with or without Supplemental Blue Light. Soil Sci. Plant Nutr..

[18] Maruyama S., Ishigami Y., Goto E. (2010). Effect of Light Period Longer than Critical Day Length after Heading on the Growth and Development of Rice under a Controlled Environment. J. Sci. High Technol. Agric..

[19] Goto E. (2011). Production of Pharmaceutical Materials Using Genetically Modified Plants Grown under Artificial Lighting. VI International Symposium on Light in Horticulture 907.

[20] Balindong J.L., Ward R.M., Liu L., Rose T.J., Pallas L.A., Ovenden B.W., Snell P.J., Waters D.L.E. (2018). Rice Grain Protein Composition Influences Instrumental Measures of Rice Cooking and Eating Quality. J. Cereal Sci..

[21] Righini I., Graamans L., van Hoogdalem M., Carpineti C., Hageraats S., van Munnen D., Elings A., de Jong R., Wang S., Meinen E. (2024). Protein Plant Factories: Production and Resource Use Efficiency of Soybean Proteins in Vertical Farming. J. Sci. Food Agric..

[22] Liu Y., Li Z.-G., Cheng H., Yang X., Li M.-Y., Liu H.-Y., Gan R.-Y., Yang Q.-C. (2025). Plant Factory Speed Breeding Significantly Shortens Rice Generation Time and Enhances Metabolic Diversity. Engineering.

[23] Maruyama S., Ishigami Y., Goto E. (2010). Effect of Nutrient Solution Concentration at the Heading Time on the Growth, Development, and Seed Storage Protein Content of Rice Plants in a Controlled Environment. Environ. Control Biol..

[24] Uchimura Y., Ogata T., Sato H., Matsue Y. (2000). Effects of Silicate Application on Lodging, Yield and Palatability of Rice Grown by Direct Sowing Culture. Jpn. J. Crop Sci..

[25] Agostinho F.B., Tubana B.S., Martins M.S., Datnoff L.E. (2017). Effect of Different Silicon Sources on Yield and Silicon Uptake of Rice Grown under Varying Phosphorus Rates. Plants.

[26] Syu C.-H., Huang C.-C., Jiang P.-Y., Chien P.-H., Wang H.-Y., Su J.-Y., Lee D.-Y. (2016). Effects of Foliar and Soil Application of Sodium Silicate on Arsenic Toxicity and Accumulation in Rice (*Oryza sativa* L.) Seedlings Grown in As-Contaminated Paddy Soils. Soil Sci. Plant Nutr..

[27] Buck G.B., Korndörfer G.H., Nolla A., Coelho L. (2008). Potassium Silicate as Foliar Spray and Rice Blast Control. J. Plant Nutr..

[28] Takahashi N., Kurata K. (2007). Relationship between Transpiration and Silica Content of the Rice Panicle under Elevated Atmospheric Carbon Dioxide Concentration. J. Agric. Meteorol..

[29] Katakura & Co-op Agri Corporation Water Silica: A New Type of Silica Fertilizer for Top-Dressing. https://www.katakuraco-op.com/site_fertilizer/products/pdf/water_silica.pdf.

[30] El-Aziz M.A.A., Elbagory M., Arafat A.A., Aboelsoud H.M., El-Nahrawy S., Khalifa T.H., Omara A.E.-D. (2025). Evaluating the Impact of Nano-Silica and Silica Hydrogel Amendments on Soil Water Retention and Crop Yield in Rice and Clover under Variable Irrigation Conditions. Agronomy.

[31] Hermans C., Hammond J.P., White P.J., Verbruggen N. (2006). How Do Plants Respond to Nutrient Shortage by Biomass Allocation?. Trends Plant Sci..

[32] Sakaigaichi T., Morita S., Abe J., Yamaguchi T. (2007). Diurnal and Phenological Changes in the Rate of Nitrogen Transportation Monitored by Bleeding in Field-Grown Rice Plants (*Oryza sativa* L.). Plant Prod. Sci..

[33] Gong H., Zhu X., Chen K., Wang S., Zhang C. (2005). Silicon Alleviates Oxidative Damage of Wheat Plants in Pots under Drought. Plant Sci..

[34] San-oh Y., Sugiyama T., Yoshita D., Ookawa T., Hirasawa T. (2006). The Effect of Planting Pattern on the Rate of Photosynthesis and Related Processes during Ripening in Rice Plants. Field Crops Res..

[35] Nakano A., Higashide T., Ahn D.-H. (2017). Relationships between Yield, Mineral Content of Fruits, and Sap Bleeding Rate in Dutch and Japanese Tomato Cultivars. Jpn. Agric. Res. Q..

[36] Ma J.F., Yamaji N. (2006). Silicon Uptake and Accumulation in Higher Plants. Trends Plant Sci..

[37] Ma J., Nishimura K., Takahashi E. (1989). Effect of Silicon on the Growth of Rice Plant at Different Growth Stages. Soil Sci. Plant Nutr..

[38] Epstein E. (1999). Silicon. Annu. Rev. Plant Physiol. Plant Mol. Biol..

[39] Lyu Y., Wu K., Zhou Y., Du J., Liu C., Wang Y., Yuan Q., Dong X., Hong Z., Fahad M. (2026). FKF1 Nuclear Condensates Control Anti-Florigen Turnover and Flowering Onset in Response to Nitrogen Availability in Monocots. Dev. Cell.

[40] Wang W., Hu B., Yuan D., Liu Y., Che R., Hu Y., Ou S., Liu Y., Zhang Z., Wang H. (2018). Expression of the Nitrate Transporter Gene OsNRT1.1A/OsNPF6.3 Confers High Yield and Early Maturation in Rice. Plant Cell.

[41] Simmonds N.W. (1995). The Relation between Yield and Protein in Cereal Grain. J. Sci. Food Agric..

[42] Triboï E., Martre P., Triboï-Blondel A.-M. (2003). Environmentally-Induced Changes in Protein Composition in Developing Grains of Wheat Are Related to Changes in Total Protein Content. J. Exp. Bot..

[43] Kudo Y., Umemoto K., Obata T., Kaneda A., Ni S.R., Mikage M., Sasaki Y., Ando H. (2023). Seasonal Variation of Alkaloids and Polyphenol in *Ephedra Sinica* Cultivated in Japan and Controlling Factors. J. Nat. Med..

[44] Ma J.F. (2004). Role of Silicon in Enhancing the Resistance of Plants to Biotic and Abiotic Stresses. Soil Sci. Plant Nutr..

[45] Voogt W., Sonneveld C., Datnoff L.E., Snyder G.H., Korndörfer G.H. (2001). Chapter 6 Silicon in Horticultural Crops Grown in Soilless Culture. Studies in Plant Science.

[46] APHA, AWWA, WEF (2021). Standard Methods for the Examination of Water and Wastewater.

