# Supplementary material for: Enhanced Seed Protein Yield in Hydroponically Grown Rice via Silica Hydrogel Application
Source: Plants (Basel). 2026 Jun 9;15(12):1775. doi: 10.3390/plants15121775 (PMC13306745; doi:10.3390/plants15121775)
Supplement: Supplementary file 1 [file plants-15-01775-s001.zip › Tables S1 and S2.pdf]

Table S1. ICP-OES operating conditions

Instrument	Agilent 5800 VDV ICP-OES
RF power	1.20 kW
Plasma gas flow rate	12 L min^{-1}
Auxiliary gas flow rate	1.0 L min^{-1}
Nebulizer gas flow rate	0.70 L min^{-1}
Sample uptake rate	12 rpm
Nebulizer	concentric SeaSpray glass nebulizer
Spray chamber	double pass glass cyclonic spray chamber
Plasma viewing mode	axial
Torch injector i.d.	2.0 mm
Stabilization time	20 s
Integration time	10 s
Replicates	3

Table S2. Candidate emission wavelengths for Si evaluated in this study

Wavelength (nm)	Transition type	Note
251.611	Atomic	1st manufacturer-recommended wavelength
288.158	Atomic	2nd manufacturer-recommended wavelength
250.690	Atomic	3rd manufacturer-recommended wavelength

Among the tested wavelengths, 288.158 nm was not selected due to its relatively high background intensity, which resulted in a lower signal-to-background ratio compared with the other wavelengths under the present analytical conditions. Although 251.611 nm showed high sensitivity, 250.690 nm was selected because it provided slightly better performance with the calibration model at the upper end of the working range.
